# On the Action of Mercury—A Paper Read before the Cayuga County Medical Society

**Published:** 1870-01

**Authors:** N. H. Eastman

**Affiliations:** The Genesee Medical College


BUFFALO

### Medical and Surgical Journal.

VOL. IX. JANUARY, 1870. No. 6.

### Original Communications.

ART. I.—*On the Action of Mercury—A Paper read before the Cayuga County Medical Society.*

BY N. H. EASTMAN, M. D.,
Of the Genesee Medical College.

It is of comparatively recent date, that the modus operandi of medicines has occupied any considerable attention of scientific men or been made the subject of special investigation by the learned portion of our profession. True, various theories have at times been propounded by speculative writers, but these have generally been so vague, so foreign from truth, and so destitute of proof or scientific demonstration, that they have either been disregarded entirely by the sober thinking portion of the profession, or if accepted as probable, have only served to mislead honest inquirers and establish systems of practice as false as they are pernicious in their tendencies and ultimate results.

Those of us who studied our profession thirty years ago, will recollect the teachings of the day embodied in the standard works on Therapeutics and Materia Medica.

The current doctrine of the schools then was, that no medicines entered the circulation, that all acted directly on the inner surface of the stomach and bowels, and indirectly and sympathetically on remote tissues, a doctrine which, even then seemed to me, though but a student and novice in physic, too unphilosophical to demand my assent, though inculcated by no less distinguished writers than Murray and Chapman.

Even now, one is led to suppose, in reading most of our late works on Materia Medica, that the majority of medicinal substances never enter the blood, but produce their effects by a direct local influence on some portion of the alimentary canal. This idea is especially carried in reference to the action of emetics, cathartics, and even astringents, when these last are given to act upon any portion of the first passages. When most of our modern authors speak of any article entering the circulation, they seem to convey the idea that it is an exception to a general rule.

Headland is, as yet, almost the only writer who has thoroughly investigated the subject of the "Action of Medicines." He has, as it were, struck out a new path in this direction, and shows most conclusively, by his own experiments as well as by those of many careful and scientific observers, that, as a rule, all medicines, after solution, are absorbed into the blood from the stomach or bowels, and subsequently produce their specific effects by actually coming in contact with the tissue on which their action is manifested, either through the walls of the capillaries, permeating such tissues, or in their exit from the blood through the various glandular structures whose function is to eliminate from the blood whatever material is unnatural to that fluid, or is required to be separated from it, either for purposes of secretion or mere excretion; that the few medicinal substances which are not absorbed, but which make their impression merely on the nervous coat of the stomach and bowels, such as irritant emetics and cathartics, are exceptions to a general rule, and produce no real specific effect on a distant tissue, but make their local impression on the part by contact, while the distant impression is the result of mere reflex action, a remote effect, as that of a revulsive, that specific emetics and cathartics, entering the blood through the walls of the stomach or bowels, or injected into the veins, cellular tissue, or serous cavities, acting by a sort of vital electric affinity, when brought in contact with the nerves or nerve centres of the stomach on the one hand, or ultimately eliminated through the intestinal glands into the bowels on the other, produce the same effect on these viscera as irritant emetic and ca thartics, brought in contact with these surfaces in their passage through the alimentary canal. Headland, in his admirable book, has but entered the threshhold of a subject that demands further investigation, and I trust his example will stimulate other laborious searchers after truth, to prosecute this interesting subject till we shall learn the modus operandi of most, if not all our medicinal agents, and thus be enabled to treat disease with far more reason and success than we hitherto have been enabled to do.

My object in this paper is to inquire into the action of Mercury, an article so varied in its preparations, so old in its application, so potent for good or for evil, and whose employment in almost every disease has its advocates on the one hand, and on the other those who regard its use, under the same circumstauces, not only useless but pernicious. Such is the diversity of opinion, even among honest and intelligent members of the profession, that the student is bewildered in his researches and compelled to grope his way in the dark, till, by long experience, he draws his conclusions from his own cautious trials and careful observation. The first question that presents itself is, does Mercury, in any or all of its forms, enter the circulat on, or does it act merely by its presence in some part of the alimentary canal? This question I regard as now settled by the most palpable experiments, since the metal has been found repeatedly by the chemist, both in the fluids and solids of the body, after having previously been introduced into the system, whether through the stomach and bowels, by inunction or fumigation; and yet, men now talk and write as though they supposed the article given by the mouth sometimes entered into the circulation, and at others, merely passed along the intestinal canal and acted simply as a local irritant, producing in this way its cathartic effects; whereas, the fact is, all forms of the mineral, except perhaps the crude metal, now seldom or never administered, before producing any real medicinal or toxical effects, are taken up in a state of solution by the capillaries of the first portion of the alimentary canal, if not before they reach even the stomach, whether the article acts subsequently as an alterative, catalytic, or simply as a cathartic in its ultimate elimination through the intestinal glands. This mode of action of mercury, as also that of most medicines is, as already stated, established by actual demonstration beyond a peradventure.

But the query still remains, how are some of the forms of mercury made soluble and thus prepared to be absorbed through the walls of the minute tissues? Some of them, as the bi-chloride, are readily dissolved in water or some other menstruum, and thus prepared for absorption, others again, as calomel, are scarcely soluble in any fluid suitable for introduction into the stomach, or in any secretion of that viscus. In regard to the crude metal, being a positive fluid already, under any ordinary temperature, and even susceptible of volatilization at any degree of heat above forty degrees below zero, Fahrenheit, I see no impossibility or even improbability of its being taken up by the tissues of any surface to which it may have been applied, whether combined or simply incorporated with any oily substance, as is usual when applied externally, or with conserve of roses as when introduced into the stomach in the form of a blue pill.

Dr. Wood and some other writers of eminence seem to suppose that calomel when taken into the stomach is slowly and partially decomposed by the chlorides of that organ, and converted into the bi-chloride of mercury, which, being in a fluid state, is easily absorbed by the gastric capillaries; but this seems hardly credible, since the action of corrosive sublimate differs very considerable from that of calomel. the former scarcely acting as a cathartic, unless given in toxic quantities, and again, it is perhaps, less likely to produce salivation when given in reference to its alterative effect, (for which it is always used,) than almost any other preparation of mercury. Headland instituted a series of experiments to ascertain the solubility of calomel, and after trying several menstrua, he discovered, as he thought, that bile was the only animal fluid that possessed this power to any appreciable extent; that a given quantity of this fluid actually dissolved a definite amount of this salt, hence he concluded that this medicine, when taken into the stomach, passed unchanged into the duodenum, when meeting with the secretion of bile, it was readily dissolved and subsequently absorbed by the capillaries ramifying on the mucous surface of this portion of the intestines With this view, he readily accounted for the difficulty of producing an effect, even with enormous quantities of the medicine, when the secretions of the liver was nearly or quite interrupted, as in cholera and some other cases, in which the article seemed to remain undissolved and passing along the intestinal track, to be finally thrown off with the excrementitious matter of the system. It must be confessed that this theory is plausible, and accounts, perhaps, as well as any other, for the solution and absorption of calomel into the blood, but yet, this is far from being satisfactorily proved.

From the speedy effects of very small doses thrown dry into the mouth or incorporated with a trifling amount of sugar, at short intervals, as in cases of cholera infantum and others, I am half inclined to think that these minute quantities are more or less perfectly dissolved by the saliva or some one of its ingredients, and absorbed directly through the mucous membrane of the mouth and fauces, and in this way enter the blood, and I have recently seen the same idea suggested by a writer in one of the foreign journals.

Whether this be the case, or the medicine in solution or in simple mixture with the secretions of the mouth, pass down into the stomach and be there dissolved and absorbed, it is remarkable how very quickly vomiting and purging are generally allayed by this mode of administration of calomel. The solubility of this article, or rather the mode by which calomel is dissolved, yet remains to be demonstrated. One thing, however, is certain, that it does enter the circulation in a state of solution from the intestinal canal, since it is pretty clearly settled that no solid substance, however minute its particles may be reduced by trituration, can pass through the basement membrane to reach the blood. Leaving the question of the solubility of calomel, and its manner of entering the circulation, I proceed to speak of its behavior, or rather the action of mercury after its ingress into the blood.

Now, I assent, and shall endeavor to prove, that mercury, however it gains admittance in the system, and in whatever way it operates, whether as as a purgative, alterative or catalytic, produces its specific effects in one of three ways, first by stimulating the capillary vessels to perform this normal function by direct contact with their walls, and thereby restoring or correcting the various secretions when suppressed or damaged; secondly, by its elimination into the intestinal canal, mainly through the intestinal glands, whereby it causes an increased flow of fluid into that channel, augments the peristaltic motion, and in this way causes purging; and lastly by working certain changes in the blood itself, and by this means counteracting certain morbific agents in that fluid, when judiciously employed, in the diseased state; and when given too freely or unnecessarily in any condition, deteriorating that fluid, by gradually depriving it of its normal amount of fibrine and other solid materials, thus depressing the general system, undermining the constitution, producing dropsies, cutaneous eruptions, and many other abnormal states that follow the reckless use of a substance so powerful for good or for evil, according as it is prudently or rashly employed. As above stated, the opinion would seem to be entertained by most medical men, if not by writers, that when mercury is given as a cathartic, it merely passes along the alimentary canal, and in this way, by local access, stimulates the mucous membrane of the intestines, and through contiguous sympathy, excites the muscular coat, and thus produces purging. Accordingly we constantly hear of giving some cathartic in combination with it or at a subsequent period to "carry it out of the system, lest it enter the circulation and cause salivation or other deleterious effects"; hence the almost universal practice of most physicians.

Now, I am willing to concede that in many cases where mercury is administered in small, and perhaps repeated doses, not sufficient to cause free catharsis, it is good practice to follow its administration with some purgative after a few hours, or to combine with it some cathartic, in the form of a pill or powder, but I apprehend the good effect in such cases results not from removing the mercurial from "the system," but rather from hurrying away, or floating off, so to speak, the increased morbid and irritating secretions poured out of the liver, and perhaps other glands, into the bowels by the stimulating action of the alterative, but not entirely removed from the intestinal canal. So far from being necessary always to follow the exhibition of mercurials with a purgative to hasten their exit from the system, it not unfrequently becomes necessary to retard their elimination by opiates to blunt the sensibility of the intestinal glands, in order to afford time for their specific alterative effect. There may be instances where the blood is overcharged with this medicine, either from necessity or recklessness, in which, cathartics may be used with advantage, for the sole purpose of hastening its elimination from the system through the alimentary canal.

Few substances can compare with this mineral in its specific stimulant effect on the capillary system, and consequently alterative action on the whole glandular structures. Hence its general use as an alterative or corrective, in all functional derangements, and hence, also, its liability to abuse. If mercury act more promptly and perceptibly on the liver, it is chiefly because it reaches this organ first, in its passages from the upper portion of the intestinal canal through the capillaries, mesenteric and portal veins on the one hand, and on the other, for the reason that this is the largest gland in the body, and consequently a change in its function is more appreciable than in some other secreting organs of less dimensions; nor do I deny that mercury is especially eliminated through the liver, and in this way produces a specific increased elimination of the biliary secretion by an established physiological law; but that the alterative effect of mercury is not limited to the liver, is proved by the well known fact that the addition of this mineral to almost any, indeed, I might say every specific glandular stimulant, materially augments the action of such stimulant, thus by combining a mercurial with a diuretic, diaphoretic, expectorant, or any other like eliminator, its action is materially and palpably increased, mainly, I apprehend, by its universal capillary stimulant effect. That mercury, even when given as a cathartic merely, produces a remedial effect by this universal excitation of capillary and glandular functions long before it purges the bowels, is evident from the amelioration of morbid symptoms appreciable to both the patient and physician. Who has not experienced in his own person, on taking an appropriate dose of this article, a sensible relief from morbid symptoms long before its evacuating effect; and what physician has not observed the great change in his patients condition after a judicious administration of calomel or some other preparation before its purgative action; the hot and dry surface becoming cool and moist, the hurried breathing growing calm, the pulse losing its frequency and sharpness, and in fine, all the abnormal symptoms giving way to the approach of returning health? Hence the advantage of a mercurial cathartic or any other purgative in the commencement of almost any disease, where a purgative is indicated, and where no idiosyncracy precludes its use. When there is needed simply an evacuating effect, as the removal of some irritant from the bowels, as crude ingesta, any other well chosen purgative will answer all the purposes, and be preferable to mercury, but when we wish to effect any change arising from congestion or oppressed or disordered secretions, in addition, or previous to an opening effect, mercury, in some form, combined with or preceding some other aperient is of the utmost utility when not contra-indicated by some peculiar idiosyncracy. All that is necessary in such cases is, to observe prudence in its administration, not to give more than is needful to accomplish the desired aim, nor repeat the medicine when not plainly indicated, for I am well aware that any substance, and especially one so potent as the one under consideration, which tends to produce any material change in the economy, when administered judiciously, cannot but prove more or less baneful, for the reason that any considerable change wrought in the healthy state must needs be deleterious, and that the unnecessarily protracted use of an article even in a morbid condition may prove equally injurious.

One way in which mercurials act as cathartic, I think, is not always recognized. This remedy, given in some abnormal states of the liver, even in a very small dose, will not unfrequently act sharply as a purgative, while the same quantity repeated after a brief period, say twenty-four or forty-eight hours, will operate far less actively, and perhaps, if given the third time after a similar interval, will cease to produce any cathartic effect. This would seem plainly to indicate that the aperient action depended immediately upon the presence of superabundant or acrid bile suddenly poured out of the liver by the action of the medicine, rather than the elimination and direct effect of the mercurial itself, and that as soon as the morbid secretion disappeared, and the healthy action of the gland became established through the stimulating and restorative effect of the mercury, the article ceased to be purgative, since the small quantity employed, though sufficient to stimulate the liver and emulge the bile ducts of their retained and morbid contents, was insufficient of itself to produce catharsis, by its elimination through the intestinal glands into the bowels. I am aware that I am here met by an objection raised by some recent experiments, and writers in the journals, that "mercury, so far from restoring or augmenting the action of the liver, actually interrupts its functions, and thereby checks or suppresses the secretion of bile, as proven by repeated experiments on living animals, as dogs, &c." and further, that "the natural yellow color of the fæces, results, not from the coloring matter of the bile, but from a peculiar secretion from the large intestines, and that the "green stools" that so generally follow the exhibition of mercury, depend on its combination with the intestinal secretions, rather than upon any morbid secretion from the liver, eliminated through the action of this alterative." These statements seem plausible, but I apprehend that the profession are not prepared to accept them as truthful till demonstrated by further observation and experiment on the human system. A doctrine so much at variance with the almost universal opinion of medical men, needs the most positive proof to establish its claims to our confidence.

It is a most palpable fact, established by daily observation, that innumerable cases occur, as jaundice, from functional disturbance of the liver, attended with ash colored stools, yellow tinge of the skin, conjunctiva and urine, symptoms, one would suppose, sufficiently indicative of the coloring matter of the bile being retained in the blood, in which a moderate exhibition of mercury is followed by a complete disappearance of all those symptoms, including a return of the yellow hue of the fæces, simultaneous with the clearing up of the whole surface and restoration of the natural amber color of the renal secretion. Again, it is equally true that in apparent torpor of the liver, or defective biliary secretion, as manifest in cholera infantum, and many other morbid conditions, a judicious use of some mercurial will be followed at first by quite dark, it may be, almost black dejections, which subsequently become greenish, and ultimately resume their natural tint, or golden yellow, before the discontinuance of this alterative. Now such facts, and they seem not to admit of a doubt, would appear sufficient to establish the old theory, that the normal color of the fæces depended on the presence of bile pigments, and that the black and green stools so often witnessed, after the exhibition of mercury, result from the abnormal hue of retained or obstructed biliary secretions, and that mercury instead of suppressing the action of the liver, positively tends to restore or augment its function, the limited and defective experiments of some modern writers to the contrary, notwithstanding. While these conclusions may be correct, I can well understand how this article, when administered untimely, or too long continued, may, by its unnecessary or over stimulant effect on the liver, derange its functions, cause morbid secretions of the organ and produce and keep up in this way, the green or chopped spinach stools that I believe not unfrequently attend the abuse of this most valuable medicine. Indeed, I can readily conceive that by a reckless employment of mercury, actual inflammation of the liver may be brought about, as well as a similar state of the salivary glands and I have no doubt but such a state has often been produced by ignorant and careless doctors. Though a wise exhibition of mercury may do much in some instances, in obviating disease by restoring the secretions of the liver, and thus preserving the purity of the blood, (for be it remembered, the liver is one of the great purifiers of that fluid, being, as it were, a great filter, occupying a central position in the system, through which all the blood of the intestinal veins, and some other abdominal viscera percolates, so to speak, before reaching the heart and general circulation,) still, I am led to conclude that much of the remedial effects of mercury, in its action on the liver, consists it in a removal of the obstruction in the hepatic capillaries by its stimulating action upon those vessels, and thus relieving abdominal congestion, necessarily resulting from partial obstructions, and producing, when not relieved in this way, cholera, diarrhea, enteritis, etc., according to the intensity and continuance of the engorgement, and that the "restoring of the secretions" is but an index to the removal of an obstructive cause, and a return to a healthy circulation, rather than the efficient cause of a restoration to the healthy condition, since the bile must needs be more or less suppressed or vitiated whenever the circulation through the liver is seriously impeded; hence we hail its re-appearance in cholera and other cases, as a sure indication of a removal of obstruction, in the portal circle, and a return to the normal condition. I have thus dwelt long on the action of mercury on the liver, for the reason that very many of our profession seem to regard this as the chief object in administering the article, that most, if not all the morbid conditions in which this mineral is found to be useful, depend on or are connected with, some abnormal state of that viscus, save, perhaps, the single exception of syphilis, while nothing, I think, is farther from the truth.

The anti-phlogistic action of this medicine seems to result from two causes, the one, and I think the first and most important is, as above stated, its specific stimulating or exciting action on the blood capillaries, or rather, I would say, the ultimate tissues themselves; for I regard the capillaries, not so much vessels, in the proper sense of that term, as mere channels permeating almost every portion of the tissues, whose walls consist, not like vessels proper, of two or more coats, but of the condensed tissues themselves, lined by a tenuous, structureless membrane, differing somewhat from that of the smallest artery or vein, precisely fitted to prevent extravasation of the blood through the tissues, on the one hand, and on the other, to admit the freest osmose of that fluid to the molecules that occupy the inconceivably minute meshes that intervene between the myriads of microscopic passages that traverse the entire structure of the animal economy.

This being the case, the mercury would appear to be brought through the circulation in intimate or direct relation with the ultimate tissues, and thus by its stimulating action on the same, to restore the depressed tone of the capillary walls, promote or recover the vital affinity that exists between them and the contained blood on which depends the capillary force, or that power which appears in part, at least, to maintain the circulation through the several structures independent of the heart's action, and in this way, to facilitate the onward passage of the blood, overcome congestion, promote absorption of effused material, or, in other words, subdue inflammation and restore the normal condition of the part; in fine, to act as a powerful antiphlogistic. That mercury is a powerful antiphlogistic, seems sufficiently proved by the observation of the whole profession, in all ages, since its first employment as a medicine. The palpable subsidence of inflammation in certain tissues, as the serous and others, under its continued use when prudently administered, the visible disappearance of effused lymph, as in iritis, during its administration, and many other like examples of its salutary effect in inflammatory affections, would seem sufficient to demonstrate the truth of the assertion, and it only remains to prove that this effect is brought about in the way I have stated. The the mineral has no direct influence on the great nerve centres, as the brain, or sympathetic ganglia, as the narcotics, nervous stimulants, and some, at least, of the bitter vegetable tonics, as quinine, appears to be proved from the fact that no immediate sensible effects follow its employment on such centres, as is the case with all the narcotics I have mentioned, whatever be the quantity administered. True, like many other mineral catalytics, its continued and injudicious use may work, and doubtless has wrought, certain destructive changes in the blood, that in the end, have been productive of various distressing nervous disorders, as tremors, epilepsy, paralysis, &c., but these are only indirect and ultimate results, common to other cathartic medicines, rather than the primary, legitimate and specific products of mercury itself, consequences that follow and depend on the deteriorated state of that fluid, whatever may have been the cause. Nor does the article appear to have any direct effect on the heart or large tissues, in-so-far as we can discover from observation, as do many other arterial stimulants and sedatives, whether acting immediately on the tissues of those organs themselves, or through their nerve tissues; nor yet can we perceive that its action is peculiar to any one glandular structure, or any portion of the fibrous tissues, as is the case with the specific eliminators, astringents, etc., but we are compelled, as it were, by the law of exclusion, to look to the capillary vessels and ultimate tissues themselves, as the peculiar seat of the action of mercury, and here we find abundant evidence of the fact. Here would seem to be the seat of all the physiological and pathological changes and modifications of the animal economy, or at least, the manifestations of such alteratives, whatever their source may be. In or through this infinite labyrinth of infinitesimal channels, all the vital processes take place as absorption, secretion, nutrition, assimilation, innervation, calorification, &c., and here, also, are manifest all the morbid changes, as congestion, inflammation, effusion, degeneration of tissues into abnormal growths, whether benign or malignant, nay, even the more purely nervous affections, as neuralgia, hyperæsthesia and anæsthesia, convulsions and paralysis, doubtless frequently, perhaps I should say always, have their origin directly from abnormal capillary circulation, whether this morbid state be caused by some direct irritation of the minute vessels or the ultimate molecules composing their parietes, or from loss of nerve force or enervation arising from defective generation of nervous fluid or influence, if you please, in the nerve centres of organized life, caused by the presence of some morbid material, as miasm, received into the blood, and thus brought in contact with such centres, causing, for the time, depression or paralysis, so to speak, of those functions of the nerve currents that go to innervate the capillary walls or the ultimate tissues of which they are constituted.

While some medicines, like opium and quinine, appear to act secondarily or ultimately on the wall of these minute vessels, by reason of their primary action on the nervous centres of animal and organic life, and in this way preserve or restore the tone of these intermediate passages through the nerve filaments that go hence to innervate them, mercury would seem to act directly and locally on the same capillary walls by actual contact with them, as it is conveyed to them by the blood in its passages through the various tissues. That a restorative action is produced by this article on the capillary vessels, thus overcoming engorgement and equalizing the circulation more or less throughout the system, seems sufficiently demonstrated to our senses, when we perceive by the feel, the wonderful change which is effected on the external surface after the exhibition of any mild mercurial, long before the medicine operates specifically as a cathartic. As above stated, the cool, soft and moist skin, the reduced frequency and hardness of the pulse, the increased action of all the emunctories, proves conclusively to my mind, that the universal effect results from no cause other than the peculiar action of the mineral on the whole capillary system of vessels, or the ultimate tissues that constitute their parietes.

That in addition to this effect of mercury, it exercises another influence when continued a sufficient length of time, I am not disposed to question. That by virtue of its cathartic effect on the blood itself, whereby that fluid becomes deteriorated, or more or less deprived of its albumen, fibrin, and normal amount of solid corpuscles, it tends powerfully to reduce its stimulating effect on the tissues in inflammatory states, in which there exists, always, perhaps, an abnormal amount of fibrin, and, at the same time, by their impoverishing process promotes absorption, not only of the morbid products, but if continued long enough, of the healthy structures, is sufficiently evident. But while this anti-phlogistic property of the mineral may be taken advantage of in sthenic cases and in good habits, in the opposite conditions it is a serious objection to its use, indeed, we are obliged often to deprive ourselves of the primary anti-phlogistic properties of this article, as I regard them, in degraded or enfeebled habits, for the very reason of the attendant evils that are liable in such cases to occur from this catalytic effect. In all such circumstances we must use the medicine, if at all, with the utmost caution, and perhaps combine with it, or administer simultaneouly, some restorative tonic, as iron and quinine, to preserve the normal integrity of the blood, while we are availing ourselves of its anti-phlogistic influence by virtue of its stimulating effects on the capillary tissues, and this principle is daily acted on all judicious practitioners, who give tonics and chalybeates in connection with aleratives when a long course of the last is requisite in protracted cases in good habits. Now, I apprehend that in giving an ordinory mercurial cathartic, or in pursuing a mild alterative course for a short period, no considerable catalytic effect is produced on the blood, nor any perceptible or lasting evil is inflicted on the fluids or solids, but the good effect we obtain depends on the temporary stimulating action of the medicine on the capillary system, as above explained, rousing the dormant vessels, giving tone to their walls, so as to cause them to retain their firmness, and thus resist the incoming blood driven into them by the heart's action, instead of yielding to the "vis a tergo" and becoming a gorger and distender, on the one hand, and on the other by promoting that vital energy of the parietes of the minute vessels, or the chemico-vital affinity between them and the blood, on which to some extent, at least, the capillary circnlation depends.

The above conclusion being correct, we readily discover with what skill and caution we should employ an article so powerful for good or for evil in a large range of diseases: that in ordinary constitutions we may safely use it, so as to obviate capillary obstruction, equalize the circulation, "restore secretions" generally, overcome congestion, and finally prevent or moderate inflammation, and thus preserve the tissues from fatal disintegration; and again by an unskilful, incautious or reckless administration of the same, we may over-stimulate and debilitate the capillaries, produce inflammation, deteriorate and ultimately destroy the vitality of the blood, interrupt the vital forces, irritate or paralyze the nerve centres, and, in fine, undermine and destroy the whole constitution; that even the smallest quantity of the article administered to certain individuals of a peculiar idosyncracy, or other person under certain circumstances, may prove disastrous, by excessive salivation, violent purg_ ing and other equally deplorable effects; but such instances are rare exceptions to the legitimate action of mercury, and should be regarded in the light of accidental results liable to accrue not only with this and all other efficient remedies, but in all the various conditions and circumstances of life.

