# Correction

**Published:** 1870-02

**Authors:** 


### Correction.

In our last JOURNAL the heading to the article contributed by Prof. N. H. Eastman, was so wholly wrong as to be incapable of correction. We can now make no amends for the mistakes, and will simply say what it should have been, and make as little mention as possible of what it actually was. It should have been : "*On the Action of Mercury. A Paper read at the Semi-Annual Meeting of the Medical Association of Central New York, held at Syracuse, December 15th,* 1868. By PROF. N. H. EASTMAN, M. D., of the Geneva Medical College."

